# Phytochemistry, anti-diabetic and antioxidant potentials of *Allium consanguineum* Kunth

**DOI:** 10.1186/s12906-022-03639-5

**Published:** 2022-06-13

**Authors:** Mater H. Mahnashi, Yahya S. Alqahtani, Ali O. Alqarni, Bandar A. Alyami, Omaish S. Alqahtani, Muhammad Saeed Jan, Fida Hussain, Zia Ul Islam, Farhat Ullah, Muhammad Ayaz, Muhammad Abbas, Umer Rashid, Abdul Sadiq

**Affiliations:** 1 Department of Pharmaceutical Chemistry, College of Pharmacy, Najran University, Najran, Saudi Arabia; 2 Department of Pharmacognosy, College of Pharmacy, Najran University, Najran, Kingdom of Saudi Arabia; 3 Department of Pharmacy, University of Swabi, Swabi, KP Pakistan; 4 Department of Pharmacy, Faculty of Biological Sciences, University of Malakand, Chakdara, KP 18000 Dir (L) Pakistan; 5 Department of Pharmacy, Abdul Wali Khan University Mardan, Mardan, KP Pakistan; 6 Department of Chemistry, COMSATS University Islamabad, Abbottabad Campus, Abbottabad, 22060 Pakistan

**Keywords:** *Allium consanguineum*, ABTS, DPPH, H_2_O_2_, α-Glucosidase α-amylase, Bioactive compounds, Molecular docking

## Abstract

**Aim:**

The study was planned to investigate the phytochemicals, antidiabetic and antioxidant studies of *A. consanguineum*.

**Methods:**

The preliminary studies were performed on crude extract and different solvent fractions. Based on the potency, the chloroform fraction was semi-purified to phyto-fractions CHF-1 – 5. Furthermore, CHF-3 was subjected to isolation of pure compounds using column chromatography. The α-glucosidase, α-amylase and antioxidant assays (DPPH, ABTS, H_2_O_2_) were performed on all samples. The in-vivo experiments on compounds 1 and 2 were also performed using oral glucose tolerance test. Docking studies were performed on α-glucosidase and α-amylase targets.

**Results:**

Among all fractions, the chloroform fraction exhibited excellent activities profile giving IC_50_ values of 824, 55, 117, 58 and 85 μg/ml against α-glucosidase, α-amylase, DPPH, ABTS and H_2_O_2_ targets respectively. Among the five semi-purified chloroform phyto-fractions (CHF-1-5), CHF-3 was the leading fraction in activities giving IC_50_ values of 85.54, 61.19 and 26.58 μg/ml against α-glucosidase, α-amylase and DPPH respectively. Based on the overall potency and physical amount of CHF-3, it was subjected to purification to get compounds 1 and 2. The two compounds were also found potent in in-vitro activities. The observed IC_50_ values for compound 1 were 7.93, 28.01 and 6.19 μg/ml against α-glucosidase, α-amylase and DPPH respectively. Similarly, the compound 2 exhibited IC_50_ of 14.63, 24.82 and 7.654 μg/ml against α-glucosidase, α-amylase and DPPH respectively. Compounds 1 and 2 were potent in decreasing the blood glucose levels in experimental animals. Compounds 1 and 2 also showed interactions with the respective enzymes with molecular docking.

**Conclusions:**

We can conclude that *A. Consanguineum* is a rich source of natural antidiabetic agents. Bioguided isolation of compound 1 and 2 showed potential inhibitions in all tested in-vitro antidiabetic targets. Further, both the compounds were also able to decrease the blood glucose levels in experimental animals.

## Background

From centuries, herbal treatments have been used as medication in different diseases. Due to their harmless effect comparatively, the natural products have gained the attention of the new scientists in the cure and management of different challenging ailments [1]. According to the American Diabetes Association (ADA), “Diabetes is a very chronic situation which is characterized by high blood sugar, poor insulin secretion and the development of insulin resistance” [2]. It is a metabolic disorder primarily due to defective problems in insulin functioning hyperglycemia arise in the body [3–5]. Numerous tissues and blood vessels are destroyed as the severity of illnesses rises, and for a long time after having diabetes, and may lead to various consequences [6], for example, complications related to heart, blood, immune system, nephron, nervous system and different type of ulcers may occur. Diabetes a well-known disease of pancreas which is further classified into different types such as type 1 diabetes mellitus (T1DM) earlier known as insulin-dependent diabetes mellitus (IDDM) or juvenile diabetes mellitus, an autoimmune disease in which beta cells of the pancreas are destroyed [7] and thus does not produce and secretes insulin and type 2 diabetes” (T2DM), also called as non-insulin-dependent diabetes mellitus (NIDDM) or adult-onset diabetes which is characterized by a decrease in insulin production [8].

Etiology of “T1DM is result of an autoimmune reaction to proteins found in the islets of Langerhans of the pancreas [9] and T2DM is caused by hereditary factors linked to insulin resistance in the body. Poor insulin secretion as a result of a variety of environmental factors such as stress, increase intake of food, obesity, physical inactivity, aging and lack of exercise [10].

Epidemiologically (diabetes is a disorder of endocrine system that affects about 6% part of the world’s population and is increasing daily) [11]. The occurrence of T1DM worldwide is continuously raising annually at rate of 3 to 5%, specifically in children of age under 5 years. T1DM accounts for more than 90% of diabetes cases in children and adolescents, with 50 to 60% of individuals diagnosed before the age of 15 years, primarily in Western world [12].

In 2010, the number of patients with T1DM was reported to 285 million people worldwide, including 90% of people with T2DM. The estimated rate of diabetes for 2030 is about 439 million worldwide, which It represents 7.7% of the global adult population (20–79) [13]. In Pakistan, the prevalence is significantly high, affecting 6.9 million people now, and is predicted to double by 2025, affecting 11.5 million people [14].

Free radicals are the cause of various issues in humans’ inclusive injury of the nervous system, atherosclerosis, ischemic coronary heart diseases, cancer, arthritis, gastritis, and reperfusion damaging of various tissues [15–17]. Free radicals from pollution, radiation, chemical agents, poisons, and deep-fried and spicy meals reduce immune system antioxidants, alter gene expression, and induce abnormal proteins. During the oxidation process, free radicals are produced in biological systems at various phases [18]. Catalase and hydroperoxidase enzymes in the human body convert hydrogen peroxide and hydroperoxides to nonradical forms and then function as natural antioxidants to combat oxidative stress [19, 20]. Drug researchers have long struggled to find a way to completely eradicate the illness using plants. Various medicinal plants are utilized as major sources of treatment in the control of “diabetes mellitus,” especially in undeveloped countries [21].

Allium is a valuable genus both economically and medicinally. *Allium consanguineum* is specie of the Amaryllidaceae family. The epidermal anatomy of the leaf of *A. consanguineum* was the most diverse. The stomatal cells in this species were the longest, measuring 6–14 μm [22]. Several species of the Allium genus were identified to have significant pharmacological activity like anti-viral, antibacterial, anti-fungal [23], anti-diabetic [24] hepatoprotective [25] anti-inflammatory, cardiovascular activities [26] and the properties in which the garlic and onion are the prominent candidates. Based on the literature survey we explore the in-vitro anti diabetic and antioxidant potentials of *A. consanguineum* in the present study.

## Material and methods

### Plants collection

In April 2015, fresh leaves and rhizomes of *Allium consanguineum* were obtained in Marghazar, District Swat, Khyber Pakhtunkhwa, Pakistan. Dr. Nasrullah, a plant taxonomist at the Botany Department in University of Malakand, identified the specie, and the given voucher specimen no H.UOM.BG.158 to the plant and then this plant *Allium consanguineum* was deposited in the University of Malakand’s Herbarium [23].

### Extraction and fractionation

*Allium consanguineum* (4 kg) was shade air-dried at normal room temperature for 2 weeks. Then, it was weighed and cut into small pieces. Powdered it in a mixer grinder and 80% methanol was used to soak the plant with occasional stirring for 20 days. After the said time period the macerated plant was filtered by using Whattman grade 1 filter paper and good quality muslin cloth. The filtrate was then concentrated by using rotary evaporator at 40 °C [27]. Finally, 290 g of crude methanolic extract (brownish colored) (7.25%) was collected. The crude methanolic extract was dissolved into 500 ml of distilled water and subsequently fractionated with 500 ml of (3 × *n*-hexane), (3 × chloroform), (3 × ethyl acetate) and finally aqueous fraction was separated [28]. The percent yields of *n*-hexane, chloroform and ethyl acetate 18 (52 g), 42 (122 g) and 23 (67 g) % respectively. The remaining amount (approximately 50 g, equivalent to 17%) diluted in water was considered as the aqueous fraction.

### Phytochemistry

The preliminary assays on the crude extract and sub-solvent fractions revealed that chloroform fraction was relatively potent compared to other fractions. Based on this information, we subjected the chloroform fraction to column chromatography [29]. Due to the overlapping or close retardation factor values, we were unable to isolate the pure compounds directly. So, initially we isolated groups of phytochemicals (semi-purified), i.e.CHF-1 to CHF-5. We subjected the semi-purified chloroform sub-fractions (CHF-1, CHF-2, CHF-3, CHF-4, and CHF-5) to the in-vitro activities. We noticed that CHF-3 was the semi-purified fraction with potent activities. We further subjected CHF-3 to small pen-column with eluting solvents n-hexane and ethyl acetate. We started the column carefully with pure n-hexane (100%) and gradually changed the polarity till the isolation of pure compounds [30].

### In-vitro α-glucosidase inhibition assay

For the example planning initial 1200 μL of phosphate buffer put in test tube and included 100 μl of each concentrate which sequential weakened going from 9, 7, and 5 mg/ml. 200 μl of glycosidase (0.5 mg/11 ml refined H_2_O) was added to the test tube in addition to 0.2 ml of substrate arrangement (glucopyranoside 15 mg/10 ml refined water) was kept at 37 °C for 20 minutes [31].

For control, 1.2 ml of PO_4_ (phosphate buffer) was added to the test tube and furthermore included 200 μl catalyst arrangement and 200 μl of substrate arrangement (C_7_H_14_O_6_ (glucopyranoside) 15 mg/10 ml refined water) just as included 800 μl of Na_2_CO_3_ arrangement and was maintained for 20 minutes at 37 °C [32]. The retention of control and test was examined at 4.05e-7 m using the accompanying equation after the recommended time:

$$\%\;\mathrm{Inhibition}=\frac{\mathrm{absorbance}\;\mathrm{of}\;\mathrm{Control}-\mathrm{absorbance}\;\mathrm{of}\;\mathrm{Sample}}{\mathrm{absorbance}\;\mathrm{of}\;\mathrm{Control}}\times100$$

### In-vitro α-amylase inhibition assay

The Bernfeld’s procedure was used to investigate in-vitro amylase inhibition [33]. The 30 μl of the testes sample is combined with 10 μl of enzyme α-amylase solution and 2 mM phosphate buffer (pH -6.9) at concentration of 10 μl or acarbose 64 mg/ml as a positive control. Ten minutes after incubation period, 40 ml of 1% starch solution were applied to initiate the reaction and then incubated for 30 minutes at 37 °C. Then 20 μl of 1 M HCl and 75 μl of iodine solution were added to the 96 well plate, the absorbance of the solution was measured at 580 nm and inhibitory response of the -amylase enzyme was calculated using the following formula.

$$\mathrm{Inhibition}\ \left(\%\right)=100\ \left(\mathrm{control}-\mathrm{test}\right)/\mathrm{control}$$

### DPPH antioxidant assay

For DPPH stock arrangement, 24 g dissolve in 100 ml of methanol and maintain at 20 °C for supplementary action. After this, 1000 micro litter was the standard absorbance set at 517 nm as utilized a control and afterward spread through aluminum foil for keeping in dark area for 1 day for the formation of radicals. At that point 0.005 g of the concentrates was dissolve into 5000 micro litter methanol as a store solution and utilized for the diverse solution development/focus for the action which are 1000, 500, 250, 125, and 62.5 μg/ml. The 2 ml solution of the examples were blended in with 2 ml of 2, 2-biphenyl-1-picryl-hydrazyl radical arrangement and saved for 15 m in obscurity zone. Following equation was utilized for the assurance of % DPPH restraint [34].

$$\%\;inhibition=\frac{A-B}A\times100$$

A for absorbance of control and B are denoted for the absorbance of sample.

### ABTS free radicals scavenging assay

The ABTS analysis of plant’s samples was performed by standard systems [35]. To plan 2, 2-azinobis (3-ethilobenzathiazoline-6-sulfunat) and each potassium dithionite solution, 7 mM of c_18_H_18_N_4_O_6_S_4_ 2, 2-azinobis (3-ethilobenzathiazoline-6-sulfunat) and 2.45 mm, potassium dithionite combination was prepared in 0.1 L of methanol were blended and put away for 1 day into faint condition to make free extremists. After that the ABTS absorbance ware utilized to 0.76 nm at 745 nm and furthermore include fifty percent (50%) methanol and 3 ml of the extract test was taken and added to the 300 μl ABTs acid solution. After amassing of the two solutions, they were saved in a hatchery at 30 °C for 15 m. The absorbance of the subsequent solution was estimated at 745 nm with photo-spectrometer again. Various weakening of ascorbic corrosive was then set up as certain controls as per a similar technique. The ABTS free extreme screening was examined by the accompanying recipe.

Formula:

$$\%\;\mathrm{scavenging}\;\mathrm{activity}=\frac{\mathrm A-\mathrm B}{\mathrm A}\mathrm X100$$

A used for the control while B represents sample absorbance.

### H_2_O_2_free radicals scavenging assay

The hydrogen peroxide antioxidant activity of test samples was evaluated by using protocol described [36]. In a 50 mM phosphate buffer with a pH of 7.4, a hydrogen peroxide solution (2 mM) was formed. 0.1 mL plant specimens were put in test tubes and their volumes were increased to 0.4 mL by adding 50 mM phosphate buffer. It was vertexed when a 0.6 mL hydrogen peroxide solution was applied to it. The absorbance of each plant sample was measured at 230 nm against the blank after 10 minutes. H_2_O_2_ scavenging activity was calculated by using the mentioned equation;

$$\mathrm{Hydrogen}\ \mathrm{peroxide}\ \mathrm{scavenging}\ \mathrm{activity}=1-\mathrm{absorbance}\ \mathrm{of}\ \mathrm{sample}/\mathrm{absorbance}\ \mathrm{of}\ \mathrm{control}\times 100$$

### Experimental animals, ethical guidelines

To perform the in-vivo studies, we obtained a written approval from Departmental Ethical Committee, University of Swabi, Pakistan via letter No; UOS-06/2021 for using Albino mice in our experiment. All methods for animals were carried out in accordance with relevant guidelines and regulations. The animals were purchased from Pharmacology section of NIH, Islamabad, Pakistan. The animals were kept in the animal house as per the standard protocols of dark and light period of 12 hours each. Animals were given excess to food and water as per the approval of Ethical Committee.

After the experimental procedure, the animals were euthanized as per the standard guidelines. Experimental animals were subjected to vapours of halothane to slowly induce anaesthesia. Afterwards, the prolong and overdose treatment with halothane cause euthanasia of experimental animals [37].

### Acute toxicity studies

For the acute toxicity studies, the albino mice were divided into six groups having random four animals of both sexes in each group. The compounds were given at concentration ranging from 200 to 1500 mg/kg intraperitoneally (i.p.). The animals were observed for 3 days after the drug administration for any unwanted effect or lethality [37].

### Induction of diabetes in mice

The diabetes was induced in mice by giving alloxan i.p. as per the standard protocols [10]. A single dose of alloxan (150 mg/kg) was given to mice and were kept on fasting for next 16 hours to induce diabetes. The animals were observed for blood glucose level after alloxan administration with the help of glucometer. The animals with random blood glucose level of more than 200 mg/dl were selected for further studies. The animals with elevated blood glucose levels were divided into six groups having five albino mice in each group. Group I was given normal saline and was negative control. Group II was given Tween 80. Group III was the positive control and was given glibenclamide. The remaining groups were given different doses of the compounds. The blood glucose levels of animals were monitored on the first, fourth, seventh and fifteenth day.

### Oral glucose tolerance test (OGTT)

In this experiment, the animals treated with control and treated groups were fasted overnight. To the albino mice, 2 mg/kg of glucose was administered against the standard glibenclamide. After the oral administration of glucose, the level of glucose in blood was checked at time intervals of 0, 30, 60 and 120 minutes for assessment of the effect of exogenously administered glucose-D on the treated albino mice. After the experiment, the OGTT was carried out for 5 days [10].

### Estimation of IC_50_ values

The Microsoft Excel software was used to compute sample concentrations that inhibited substrate hydrolysis by 50% (IC_50_). The IC_50_s were determined using the same approach in free radical assays such as DPPH, ABTS, and H_2_O_2_.

### Statistical data analysis

The findings can be stated as mean ± SEM. Statistical analyzes was carried out using variance analysis (ANOVA). Differences with the *P* < 0.05 values was measured significant for all experiments. Samples were considered Statistically significant when *P* values was less than 0.05 [35].

### Docking studies

We have performed docking studies of two isolated compounds in the binding sites of α-amylase and α-glucosidase [38]. Molecular Operating Environment 2016 package was used for this purpose [39]. Crystal structure of α-amylase was obtained from protein data bank. The accession code of the obtained α-amylase was 4 W93. While for α-glucosidase, we used the constructed model from our previous studies for docking. Our previously reported protocols were used for preparation of 3-D structures of compounds and obtained enzymes, validation of docking procedure and docking simulations [40, 41]. Finally, binding orientations and interaction plots of the docked compounds were analysed by using Discovery Studio Visualizer [42].

## Results

### Phytochemistry and plant samples

**Fig. 1:**
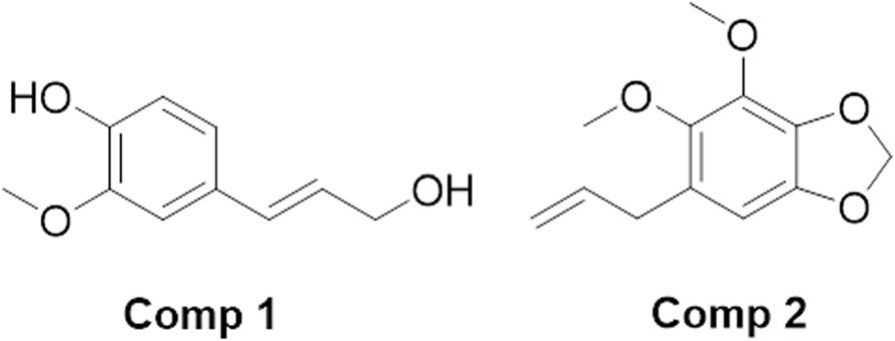
The structures of isolated compounds from *Allium consanguineum*

In this designed work, initially we screened the crude extract and solvent fractions of *A. consanguineum* for antidiabetic targets. In the preliminary screening, we observed that chloroform fraction was better in activities compared to all other fractions. So, we further headed over for purification. In the initial phase of purification, we obtained semi-purified phytochemicals with label as CHF-1, CHF-2, CHF-3, CHF-4 and CHF-5. Those unidentified groups of phytochemicals were also subjected to the in-vitro studies. Based on the in-vitro antidiabetic potential, we further subjected CHF-5 for purification. In the final stage, we were able to get two of the purified bioactive compounds 1 and 2 as shown in Fig. 1.

### Compound 1 (Coniferol)

The chemical name of compounds 1 is (*E*)-4-(3-hydroxyprop-1-en-1-yl)-2-methoxyphenol. The structures of the compounds were determined with NMR and MS analyses. The compound 1 showed molecular ion peak at 180 (58%). Furthermore, the fragmentation pattern was 151 (15%), 137 (100%), 124 (57%), 109 (19%), 91 (42%), 77 (20%), 65(21%) and 42 (5%). The ^1^H NMR of compound 1 showed a singlet at chemical shift of 3.81 ppm which represents the methoxy group attached to the benzene ring. The methylene (−CH_2_-) appeared next to the singlet in the form of a triplet at chemical shift of 4.11 ppm. The hydroxyl group attached to the methylene unit also gave a singlet at 4.53 ppm. The two unsaturated aliphatic protons appeared between 6.00 and 6.32 ppm as a doublet and multiplet. The proton at β position appeared in the form of multiplet from 6.00 to 6.13 ppm. The other proton at the α position to the aromatic ring gave a doublet at 6.32 with large coupling constant value (*J* = 17.65) due to the trans stereochemistry. The aromatic region gave two doublets at 6.92 and 7.06 ppm which is obvious from the structure. Similarly, a singlet of one proton also appeared in the downfield region of 7.17 ppm which represents the CH at ortho position to methoxy group. The OH group attached to the benzene ring appeared in the downfield region at 8.79 ppm.

### Compound 2 (dillapiole)

The chemical name of compound 2 is 6-allyl-4,5-dimethoxybenzo[d ][1,3]dioxole. The MS spectrum of compound 2 showed molecular ion peak at 222 (100%) with fragmentation pattern of 207 (20%), 191 (8%), 177 (41%), 161 (6%), 149 (126%), 134 (12%), 106 (22%), 91 (18%), 77 (28%) and 51 (16%). The most dominant peak of compound 2 was the methylene unit (−CH_2_-) of dioxolane in ^1^H NMR spectrum. These two protons appeared as singlet in chemical shift of 5.89 ppm. Similarly, the two methoxy groups appeared as two distinct singlets (three protons each) at chemical shift of 3.72 and 3.74 ppm. The single aromatic proton gave a singlet at 6.93 ppm. The two benzylic methylene protons (−CH_2_-) appeared as doublet with coupling constant value of 3.8 Hz at 3.38 ppm. The three protons attached to the alkene unit (CH_2_ = CH-) appeared between 3.5 and 6.0 ppm as per the standard chemical shift values. Of these three protons, the single proton (CH_2_ = CH-CH_2_-) gave multiplet due to the attached neighboring chemically different protons. Being diastereotopic in nature, the two protons (CH_2_ = CH-) appeared in shape of two distinct doublet-of-doublets at 4.76 and 5.18 ppm respectively.

#### In-vitro antidiabetic activities on crude extracts and solvent fractions

##### α-Glucosidase inhibition assay

As α-glucosidaseis an enzyme responsible for diabetes, and therefore has been used in phyto-medicine for assessment of anti-diabetic activities in plants. Analysis of plant samples against α-Glucosidase revealed that the highest scavenging effect is shown by the chloroform fraction, which shows 53.67 ± 0.67, 44.51 ± 1.67, 36.38 ± 1.15, 29.45 ± 0.83 and 19.73 ± 0.69 activity at the concentration of 1000 μg/ml, 500 μg/ml, 250 μg/ml, 125 μg/ml and 62.5 μg/ml respectively with the IC_50_ value 824. The standard drug acarbose exhibited 75.50 ± 1.00, 70.00 ± 0.00, 64.50 ± 0.57, 56.53 ± 0.55 and 51.63 ± 0.33% inhibitionat 1000–62.5 μg/ml respectively with 50 IC_50_ value. The lowest α-Glucosidase scavenging activity was recorded for *n*-Hexane fraction possessing the IC_50_ value 1248 μg/ml. The order of α-Glucosidase scavenging activity for all the test samples was Chloroform> Ethyl acetate>Methanolic>Aqueous>*n*-Hexane (Table 1).

**Table 1:** Alpha glucosidase inhibitory potentials of *Allium consanguineum*

Samples	Conc (μg/ml)	Percent Inhibition (mean ± SEM)	IC_**50**_ (μg/ml)
**Methanolic extract**	1000	41.10 ± 0.65***	1123
	500	37.33 ± 0.33***	
	250	17.76 ± 0.40***	
	125	15.00 ± 0.58***	
	62.5	07.00 ± 1.00***	
***n*-Hexane fraction**	1000	32.77 ± 0.76***	1248
	500	26.67 ± 0.88***	
	250	19.93 ± 0.49***	
	125	16.33 ± 0.88***	
	62.5	06.17 ± 0.13***	
**Chloroform fraction**	1000	53.67 ± 0.67***	824
	500	44.51 ± 1.67***	
	250	36.38 ± 1.15***	
	125	29.45 ± 0.83***	
	62.5	19.73 ± 0.69***	
**Ethyl acetate fraction**	1000	47.54 ± 0.44***	1076
	500	36.90 ± 1.05***	
	250	24.53 ± 0.55***	
	125	20.03 ± 1.53***	
	62.5	13.63 ± 0.33***	
**Aqueous fraction**	1000	31.50 ± 0.69***	1219
	500	22.53 ± 1.34***	
	250	17.26 ± 0.50***	
	125	11.45 ± 1.12***	
	62.5	06.86 ± 0.67***	
**Acarbose**	1000	75.50 ± 1.00	50
	500	70.00 ± 0.00	
	250	64.50 ± 0.57	
	125	56.53 ± 0.55	
	62.5	51.63 ± 0.33	

All values are taken as Mean ± SEM (*n* = 3). The *P* values less than 0.05 were considered as statistically significant. Values significantly different in comparison to standard drug i.e.** = *P* < 0.01 and *** = *P* < 0.001

##### α-Amylase inhibition assay

In the α-Amylase inhibition assay the highest activity was shown by chloroform fraction. They displayed 84.76 ± 0.61, 81.36 ± 1.31, 66.27 ± 1.06, 61.17 ± 1.30 and 55.82 ± 0.95% inhibitions at concentration from (1000–62.5 μg/mL) with IC_50_55μg/ml. ethyl acetate fraction showedsecond highest activity resulting 67.60 ± 1.63, 64.42 ± 1.89, 58.25 ± 1.40, 51.10 ± 0.60 and 46.68 ± 0.22 with IC_50_ of 100 μg/ml. Acarbose as standard drug show activity 87.49 ± 0.60, 76.28 ± 1.94, 70.08 ± 1.04, 65.37 ± 0.56 and 62.93 ± 1.73% inhibition with IC_50_ value 35 μg/ml against α-amylase (Table 2). All the other fractions in this assay displayed good to moderate inhibition.

**Table 2:** Alpha amylase inhibitory potentials of *Allium consanguineum*

Samples	Conc (μg/ml)	Percent Inhibition (mean ± SEM)	IC_**50**_ (μg/ml)
**Methanolic extract**	1000	75.29 ± 0.64***	285
	500	64.72 ± 0.89***	
	250	47.44 ± 0.86***	
	125	42.78 ± 0.45***	
	62.5	29.88 ± 0.89***	
***n*-Hexane fraction**	1000	79.95 ± 2.01**	310
	500	58.89 ± 4.82***	
	250	46.22 ± 1.28***	
	125	40.51 ± 0.54***	
	62.5	35.40 ± 0.82***	
**Chloroform fraction**	1000	84.76 ± 0.61*	55
	500	81.36 ± 1.31***	
	250	66.27 ± 1.06***	
	125	61.17 ± 1.30***	
	62.5	55.82 ± 0.95***	
**Ethyl acetate fraction**	1000	67.60 ± 1.63***	100
	500	64.42 ± 1.89***	
	250	58.25 ± 1.40***	
	125	51.10 ± 0.60***	
	62.5	46.68 ± 0.22***	
**Aqueous fraction**	1000	75.52 ± 3.28***	240
	500	55.59 ± 3.28***	
	250	50.83 ± 1.21***	
	125	45.87 ± 0.85***	
	62.5	34.54 ± 0.60***	
**Acarbose**	1000	87.49 ± 0.60	20.20
	500	76.28 ± 1.94	
	250	70.08 ± 1.04	
	125	65.37 ± 0.56	
	62.5	62.93 ± 1.73	

All values are taken as Mean ± SEM (*n* = 3). The *P* values less than 0.05 were considered as statistically significant. Values significantly different in comparison to standard drug i.e.** = *P* < 0.01 and *** = *P* < 0.001

##### Antioxidant results

The antioxidant results of the three methods are summarized in Table 3. In the DPPH scavenging activity, the methanolic extract and different fractions such as*n*-hexane, chloroform, ethyl acetate, aqueous were tested at various concentrations of 62.5 μg/ml, 125 μg/ml, 250 μg/ml, 500 μg/ml and 1000 μg/ml caused a percent radical scavenging of 71.16 ± 0.44, 63.06 ± 0.63, 52.66 ± 0.88, 44.83 ± 0.72, 38.50 ± 0.28, (IC_50_ 168 μg/ml),65.26 ± 0.37, 54.33 ± 0.88, 45.73 ± 0.37, 34.66 ± 1.20, 29.83 ± 0.72 (IC_50_ 356 μg/ml),68.66 ± 0.88, 59.10 ± 0.49, 49.40 ± 0.94, 41.56 ± 0.86,36.10 ± 0.49 (IC_50_ 117 μg/ml),74.06 ± 0.52, 66.66 ± 0.88, 59.50 ± 0.28, 52.16 ± 0.72, 47.66 ± 1.20(IC_50_ 265 μg/ml), 56.63 ± 0.68, 47.16 ± 0.44, 41.73 ± 1.15, 33.40 ± 0.83, 26.66 ± 0.88 (IC_50_ 650 μg/ml)respectively. The inhibition of all fractions was significant at the highest concentration when compared to the positive control as shown in Table 3.

**Table 3:** Antioxidant results of different fractions of *Allium consanguineum.*

Samples	Conc (μg/ml)	DPPH assay		ABTS assay		H_**2**_O_**2**_ assay	
		Percent Inhibition (mean ± SEM)	IC_**50**_ (μg/ml)	Percent Inhibition (mean ± SEM)	IC_**50**_ (μg/ml)	Percent Inhibition (mean ± SEM)	IC_**50**_ (μg/ml)
**Methanolic extract**	1000	71.16 ± 0.44***	168	68.00 ± 0.57***	148	63.33 ± 0.88***	277
	500	63.06 ± 0.63***		64.63 ± 0.48***		56.08 ± 0.86***	
	250	52.66 ± 0.88***		57.96 ± 0.12***		49.00 ± 0.57***	
	125	44.83 ± 0.72***		48.66 ± 0.88***		43.01 ± 1.21***	
	62.5	38.50 ± 0.28***		43.26 ± 0.32***		37.43 ± 0.46***	
***n*-Hexane fraction**	1000	65.26 ± 0.37***	356	51.07 ± 1.02***	937	44.96 ± 0.90***	1408
	500	54.33 ± 0.88***		45.33 ± 0.33***		37.43 ± 0.66***	
	250	45.73 ± 0.37***		37.20 ± 0.91***		32.66 ± 0.33***	
	125	34.66 ± 1.20***		33.46 ± 0.54***		28.46 ± 1.21***	
	62.5	29.83 ± 0.72***		28.63 ± 0.48***		24.06 ± 0.49***	
**Chloroform fraction**	1000	68.66 ± 0.88***	117	77.43 ± 0.61**	58	73.83 ± 0.95***	85
	500	59.10 ± 0.49***		73.26 ± 0.32**		65.05 ± 0.62***	
	250	49.40 ± 0.94***		65.56 ± 0.40***		61.33 ± 0.33***	
	125	41.56 ± 0.86***		59.03 ± 0.38***		53.06 ± 0.49***	
	62.5	36.10 ± 0.49***		55.46 ± 0.54***		48.66 ± 0.33***	
**Ethyl acetate fraction**	1000	74.06 ± 0.52***	265	68.46 ± 0.50***	186	67.83 ± 1.05***	194
	500	66.66 ± 0.88***		54.16 ± 0.85***		60.26 ± 0.93***	
	250	59.50 ± 0.28***		52.63 ± 0.52***		53.66 ± 0.66***	
	125	52.16 ± 0.72***		47.02 ± 1.11***		44.33 ± 0.84***	
	62.5	47.66 ± 1.20***		42.03 ± 0.38***		39.01 ± 1.21***	
**Aqueous fraction**	1000	56.63 ± 0.68***	650	46.66 ± 0.33***	1354	47.02 ± 0.90***	1143
	500	47.16 ± 0.44***		41.00 ± 0.57***		37.26 ± 0.40***	
	250	41.73 ± 1.15***		32.66 ± 0.58***		32.46 ± 0.52***	
	125	33.40 ± 0.83***		29.13 ± 0.88***		27.73 ± 1.05***	
	62.5	26.66 ± 0.88***		25.20 ± 0.91***		23.33 ± 0.84***	
**Ascorbic acid**	1000	92.55 ± 0.35	< 0.1	87.83 ± 0.29	< 0.1	82.43 ± 0.52	12.63
	500	87.84 ± 0.26		86.66 ± 0.66		74.03 ± 0.64	
	250	81.33 ± 0.88		81.00 ± 1.06		71.56 ± 0.49	
	125	76.54 ± 0.54		79.03 ± 0.87		67.05 ± 0.49	
	62.5	72.67 ± 0.19		76.16 ± 0.85		63.26 ± 0.93	

All values are taken as Mean ± SEM (*n* = 3). The *P* values less than 0.05 were considered as statistically significant. Values significantly different in comparison to standard drug i.e.* = *P* < 0.05, ** = *P* < 0.01 and *** = *P* < 0.001

In comparison to the DPPH results, plant extracts showed significant ABTS free radical scavenging activity. Using this assay, methanolic, *n*-hexane, chloroform, ethyl acetate, aqueous fractions at tested concentrations (62.5–1000 μg/ml) proved a percent inhibition of 68.00 ± 0.57, 64.63 ± 0.48, 57.96 ± 0.12, 48.66 ± 0.88, 43.26 ± 0.32 (IC_50_ 148 μg/ml), 51.07 ± 1.02, 45.33 ± 0.33, 37.20 ± 0.91, 33.46 ± 0.54, 28.63 ± 0.48 (IC_50_ 937 μg/ml), 77.43 ± 0.61, 73.26 ± 0.32, 65.56 ± 0.40, 59.03 ± 0.38, 55.46 ± 0.54 (IC_50_ 58 μg/ml), 68.46 ± 0.50, 54.16 ± 0.85, 52.63 ± 0.52, 47.02 ± 1.11, 42.03 ± 0.38 (IC_50_ 186 μg/ml), 46.66 ± 0.33, 41.00 ± 0.57, 32.66 ± 0.58, 29.13 ± 0.88, 25.20 ± 0.91, (IC_50_1354 μg/ml)free radicals respectively (Table 3). Ascorbic acid (positive control) inhibition was (IC_50_ < 0.1).

Against hydrogen peroxide, the scavenging abilities of different fractions are assessed. Hydrogen peroxide is nonreactive at low concentrations, but at large concentrations it is harmful to living cells and transforms into hydroxyl radicals, which are free radicals. The hydroxyl free radical may easily penetrate cell membranes and react with most biomolecules in live cells, causing tissue damage, cancer, and cell death. To protect life, the hydroxyl free radical must be eradicated. The plant samples assayed for antioxidant activity against Hydrogen peroxide revealed the highest antioxidant effect for Chloroform fraction and ethyl acetate fraction which possessed scavenging effect 73.83 ± 0.95, 65.05 ± 0.62, 61.33 ± 0.33, 53.06 ± 0.49, 48.66 ± 0.33 and 67.83 ± 1.05, 60.26 ± 0.93, 53.66 ± 0.66, 44.33 ± 0.84, 39.01 ± 1.21 at the concentration of 1000, 500, 250, 125 and 62.5 μg/ml with IC_50_ 85 and 194. Among all fractions chloroform fraction showed comparable results with standard drug ascorbic acid having the IC_50_12.63 (Table 3).

#### In-vitro antidiabetic activities on semi-purified phytochemicals (Chf-1 to Chf-5)

##### α-Glucosidase results

The α-glucosidase results of the semi-purified chloroform fractions are summarized in Table 4. We isolated different phytochemicals’ groups (CHF-1, CHF-2, CHF-3, CHF-4, CHF-5) and subjected to the in-vitro activities. Among the phytochemical groups, CHF-3was found to be the most potent giving IC_50_ value of 85.54 μg/ml. This IC_50_ value was compared with the standard acarbose (IC_50_ of 12.57 μg/ml). The potent phyto-fraction (CHF-3) gave percent inhibitions of 71.23, 65.45, 61.90, 54.00 and 45.90% at concentrations of 1000, 500, 250, 125 and 62.5 μg/ml. All the other phyto-fractions were also very good in alpha glucosidase inhibitions. The observed IC_50_ values for CHF-1, CHF-2, CHF-4 and CHF-5 were 155.39, 87.45, 316.28 and 139.72 μg/ml. The activities of all the phyto-fractions were compared with the standard acarbose at the same tested concentrations and observed an IC_50_ value of 12.57 μg/ml.

**Table 4:** Alpha glucosidase inhibitory potentials of semi-purified phytochemicals of *Allium consanguineum*

Samples	Conc (μg/ml)	Percent Inhibition (mean ± SEM)	IC_**50**_ (μg/ml)
**CHF-1**	1000	85.53 ± 1.10^ns^	155.39
	500	63.33 ± 1.90***	
	250	53.33 ± 3.83***	
	125	44.43 ± 1.10***	
	62.5	40.00 ± 0.00***	
**CHF-2**	1000	78.42 ± 0.43***	87.45
	500	73.76 ± 0.71***	
	250	68.56 ± 1.06***	
	125	55.03 ± 0.35***	
	62.5	43.08 ± 0.47***	
**CHF-3**	1000	71.23 ± 0.22***	85.54
	500	65.45 ± 0.90***	
	250	61.90 ± 0.60***	
	125	54.00 ± 0.30***	
	62.5	45.90 ± 0.45***	
**CHF-4**	1000	64.96 ± 0.32***	316.28
	500	59.74 ± 1.13***	
	250	47.33 ± 0.29***	
	125	37.11 ± 0.06***	
	62.5	23.68 ± 0.05***	
**CHF-5**	1000	67.00 ± 0.57***	139.72
	500	61.00 ± 1.15***	
	250	55.67 ± 0.67***	
	125	49.00 ± 0.57***	
	62.5	42.67 ± 0.33***	
**Acarbose**	1000	89.50 ± 1.00	12.57
	500	83.00 ± 0.00	
	250	77.50 ± 0.57	
	125	73.53 ± 0.55	
	62.5	67.63 ± 0.33	

All values are taken as Mean ± SEM (*n* = 3). The *P* values less than 0.05 were considered as statistically significant. Values significantly different in comparison to standard drug i.e.** = *P* < 0.01 and *** = *P* < 0.001

##### α-Amylase inhibition results

The alpha amylase inhibition results of the semi-purified phyto-fractions (CHF-1 to CHF-5) are provided in Table 5. In almost in a similar way, the CHF-3 and CHF-5 were equally potent phyto-fractions giving IC_50_ values of 61.19 and 58.34 μg/ml respectively. At maximum concentration, CHF-3 and CHF-5 gave percent inhibitions of 76.76 and 74.46% respectively. The observed IC_50_ values for phyto-fractions CHF-1, CHF-2 and CHF-4 were 83.03, 78.80 and 99.19 μg/ml respectively. In comparison, the standard drug gave IC_50_ value of 13.13 μg/ml.

**Table 5:** Alpha amylase inhibitory potentials of semi-purified phytochemicals from *Allium consanguineum*

Samples	Conc (μg/ml)	Percent Inhibition (mean ± SEM)	IC_**50**_ (μg/ml)
**CHF-1**	1000	75.22 ± 0.47***	83.03
	500	79.62 ± 0.36***	
	250	61.75 ± 0.58***	
	125	57.53 ± 0.71***	
	62.5	43.48 ± 0.50***	
**CHF-2**	1000	71.53 ± 0.49***	78.80
	500	68.46 ± 0.63***	
	250	63.31 ± 0.57***	
	125	60.34 ± 0.65***	
	62.5	42.47 ± 0.55***	
**CHF-3**	1000	76.76 ± 0.66***	61.19
	500	71.32 ± 1.11***	
	250	65.56 ± 1.04***	
	125	57.22 ± 0.57***	
	62.5	49.98 ± 0.65***	
**CHF-4**	1000	69.62 ± 0.70***	99.19
	500	63.51 ± 0.59***	
	250	60.44 ± 0.58***	
	125	55.63 ± 0.64***	
	62.5	42.45 ± 0.55***	
**CHF-5**	1000	74.46 ± 0.60***	58.34
	500	70.68 ± 0.60***	
	250	65.85 ± 0.56***	
	125	61.64 ± 0.75***	
	62.5	46.81 ± 0.80***	
**Acarbose**	1000	87.49 ± 0.60	13.13
	500	82.28 ± 1.94	
	250	77.08 ± 1.04	
	125	72.37 ± 0.56	
	62.5	65.93 ± 1.73	

All values are taken as Mean ± SEM (*n* = 3). The P values less than 0.05 were considered as statistically significant. Values significantly different in comparison to standard drug i.e.** = *P* < 0.01 and *** = *P* < 0.001

##### DPPH anti-radicals assay

Obviously, it can be concluded from the published literature related to the free radicals’ role in antidiabetic that the antioxidant protects the beta cell, and thus can be helpful as supplement in antidiabetic treatment. The antioxidant results using DPPH free radicals are summarized in Table 6. Comparatively, the phyto-fractions gave potent IC_50_ values than alpha glucosidase and amylase. The CHF-3 was again observed to be most potent giving IC_50_ value of 26.58 μg/ml. This phyto-fraction gave inhibitions of 81.85, 76.59, 69.75, 64.47 and 59.12% at 1000, 500, 250, 125 and 62.5 μg/ml respectively. The potencies of other phyto-fractions were in an order of CHF-5 > CHF-4 > CHF-2 > CHF-1 as obvious in Table 6. The standard drug ascorbic acid was dominant with IC_50_ value of 4.32 μg/ml.

**Table 6:** Percent DPPH free radicals scavenging results of semi-purified phytochemicals from *Allium consanguineum*

Samples	Conc (μg/ml)	Percent Scavenging (mean ± SEM)	IC_**50**_ (μg/ml)
**CHF-1**	1000	69.47 ± 0.22***	91.07
	500	63.94 ± 0.45***	
	250	57.61 ± 1.70***	
	125	53.64 ± 0.16***	
	62.5	46.52 ± 0.38***	
**CHF-2**	1000	74.42 ± 0.68***	78.12
	500	66.22 ± 0.73***	
	250	61.00 ± 0.33***	
	125	56.44 ± 0.63***	
	62.5	46.96 ± 0.42***	
**CHF-3**	1000	81.85 ± 0.18***	26.58
	500	76.59 ± 0.30***	
	250	69.75 ± 0.14***	
	125	64.47 ± 0.49***	
	62.5	59.12 ± 0.34***	
**CHF-4**	1000	76.72 ± 0.66***	61.50
	500	71.36 ± 1.11***	
	250	65.56 ± 1.04***	
	125	57.28 ± 0.57***	
	62.5	49.94 ± 0.65***	
**CHF-5**	1000	73.39 ± 0.60***	52.35
	500	67.39 ± 0.49***	
	250	61.36 ± 0.49***	
	125	57.34 ± 0.55***	
	62.5	51.90 ± 1.16***	
**Ascorbic acid**	1000	92.55 ± 0.35	4.32
	500	87.84 ± 0.26	
	250	83.33 ± 0.88	
	125	80.54 ± 0.54	
	62.5	75.67 ± 0.19	

All values are taken as Mean ± SEM (*n* = 3). The P values less than 0.05 were considered as statistically significant. Values significantly different in comparison to standard drug i.e.* = *P* < 0.05, ** = *P* < 0.01 and *** = *P* < 0.001

#### In-vitro antidiabetic activities on isolated compounds 1 & 2

Based on the potency of phyto-fraction CHF-3 in tested in-vitro assays, this fraction was further subjected to isolate compounds 1 and 2. The two compounds were also tested against alpha glucosidase, alpha amylase and DPPH targets (Table 7). The pure compounds were found to be most potent in all the three assays. In alpha glucosidase assay, compounds 1 and 2 exhibited IC_50_ values of 7.93 and 14.63 μg/ml respectively in comparison to the standard acarbose (IC_50_ 12.57 μg/ml).Similarly, in alpha amylase target, the compounds 1 and 2 were almost equally potent giving IC_50_ values of 28.01 and 24.82 μg/ml respectively. To check the possible supplementary role of isolated compounds in inhibiting the free radicals, the DPPH assay was also accompanied herein. The compound 1 was potent antioxidant compared to 2 giving IC_50_ value of 6.19 μg/ml.

**Table 7:** Alpha glucosidase, alpha amylase and DPPH inhibitory potentials of isolated compounds **1** and **2**

Samples	Conc (μg/ml)	Alpha glucosidase		Alpha amylase		DPPH	
		Percent Inhibition (mean ± SEM)	IC_**50**_ (μg/ml)	Percent Inhibition (mean ± SEM)	IC_**50**_ (μg/ml)	Percent Inhibition (mean ± SEM)	IC_**50**_ (μg/ml)
**Comp 1**	1000	85.37 ± 0.08^ns^	7.93	85.23 ± 0.22^ns^	28.01	89.24 ± 0.79^**^	6.19
	500	81.65 ± 0.44^ns^		79.45 ± 0.90^ns^		85.43 ± 1.39^*^	
	250	76.35 ± 1.42^ns^		74.90 ± 0.60^ns^		80.48 ± 0.25^**^	
	125	72.21 ± 0.39^ns^		67.00 ± 0.30*		76.47 ± 0.04^***^	
	62.5	67.66 ± 0.78^ns^		58.90 ± 0.45**		71.47 ± 0.44^***^	
**Comp 2**	1000	77.82 ± 0.86***	14.63	81.61 ± 1.70**	24.82	87.62 ± 0.58***	7.64
	500	72.67 ± 0.67***		74.33 ± 1.20***		82.35 ± 0.23***	
	250	67.46 ± 1.67***		68.33 ± 0.49***		78.36 ± 0.84***	
	125	63.58 ± 0.92***		64.70 ± 1.60**		73.62 ± 0.25***	
	62.5	61.36 ± 1.15**		59.33 ± 0.67**		69.16 ± 0.16***	
**Acarbose**	1000	89.50 ± 1.00	12.57	87.49 ± 0.60	13.13		
	500	83.00 ± 0.00		82.28 ± 1.94			
	250	77.50 ± 0.57		77.08 ± 1.04		–	–
	125	73.53 ± 0.55		72.37 ± 0.56			
	62.5	67.63 ± 0.33		65.93 ± 1.73			
**Ascorbic acid**						92.55 ± 0.35	4.32
						87.84 ± 0.26	
	–	–	–	–	–	83.33 ± 0.88	
						80.54 ± 0.54	
						75.67 ± 0.19	

All values are taken as Mean ± SEM (*n* = 3). The P values less than 0.05 were considered as statistically significant. Values significantly different in comparison to standard drug i.e.** = *P* < 0.01 and *** = *P* < 0.001

### In-vivo antidiabetic results

#### Acute toxicity study results

The doses were in the range of 200–1500 mg/kg body weight were selected on the basis of the acute toxicity series finding LD_0_ to LD_100_. For the isolated compounds 1 & 2, the detailed dosing regimen is presented in Table 8.

**Table 8:** Acute-toxicity studies with tested synthesized compounds.

Groups	Animals	Compounds,(mg/kg) 1 & 2
**1**	6	200
**2**	6	300
**3**	6	400
**4**	6	500
**5**	6	1000
**6**	6	1500

*n* = 6 per group

Initially, all the animals under acute toxicity observations were checked for behavioral and other effects. The observations were continued for 3 days. It was observed that no abnormalities were found in animals. So, a dose of 1000 mg was considered to be safe in experimental animals as this was roughly considered as the lethal dose (LD_50_).

### In-vivo anti-diabetic results of compounds 1 and 2 in albino mice

The compounds 1 and 2 were good enough in lowering the blood glucose levels in experimental albino. The observed decrease in blood glucose level by group treated with compounds 1 were 119, 205, 47, 37 and 36 mg/dl at concentration ranging from 500 to 31.25 μg/kg respectively in 15 days experiments. Similarly, the decrease in blood glucose levels with compound 2 were 201, 112, 68, 59 and 33 mg/dl at concentrations ranging from 500 to 31.25 μg/kg respectively as shown in Table 9. The decrease in blood glucose level on 15 days with glibenclamide was 274 mg/dl.

**Table 9:** In-vivo results of synthesized compounds against the standard drug

Groups		Dose μg/kg	Blood Glucose Level(mg/dl)					Decrease in Blood Glucose (mg/dl)	Change in body weight (gm)
			0 day	4th day	7th day	10th day	15th day		
Diabetic control		0.35 ml	477	482	502	513	525	-48	−13.6
Normal control saline		0.35	124	110	102	95	91	33	–
Glibenclamide		0.5	472	302	256	211	198	274	+ 10.1
**Comp 1**	1	500	411	387	371	311	292	119	+ 7.2
	2	250	420	394	345	217	215	205	+ 5.6
	3	125	441	433	414	402	394	47	+ 4.3
	4	62.5	381	372	364	352	344	37	+ 4.1
	5	31.25	415	407	398	381	379	36	+ 2.1
**Comp 2**	1	500	457	403	362	304	256	201	+ 7.3
	2	250	467	462	444	408	355	112	+ 3.3
	3	125	436	430	421	393	368	68	−3.5
	4	62.5	385	358	348	335	326	59	−4.4
	5	31.25	451	447	430	424	418	33	−6.6

### Oral glucose tolerance test

The observations of oral glucose tolerance test are summarized in Table 10. In this assay, the result of albino mice treated with compound 1 were good and was 160.1 g/dl. Similarly, compound 2 showed 151.6 mg/dl. In comparison to our compounds, the standard glibenclamide showed 140.7 mg/dl after 120 minutes.

**Table 10:** Oral Glucose tolerance test results (OGTT)

Treatment	Conc/route	OGTT (mg/dl)			
		0 minutes	30 minutes	60 minutes	120 minutes
**Group-I (Tween80)**	Oral	212.7	230.1	252.5	295.5
**Group-II (GB)**	Oral	151.3	176.5	214.1	140.7
**Comp 1**	Oral	165.6	194.6	211.4	160.1
**Comp 2**	Oral	163.5	189.4	214.6	151.6

### Docking studies

We have performed docking studies of two isolated compounds in the binding sites of α-amylase and α-glucosidase. Molecular Operating Environment 2016 package was used for this purpose [43]. Crystal structure of α-amylase was obtained from protein data bank. The accession code of the obtained α-amylase was 4 W93. While for α-glucosidase, we used the constructed model from our previous studies for docking. Prepared structures of isolated compounds and enzymes were used for docking.

**Fig. 2:**
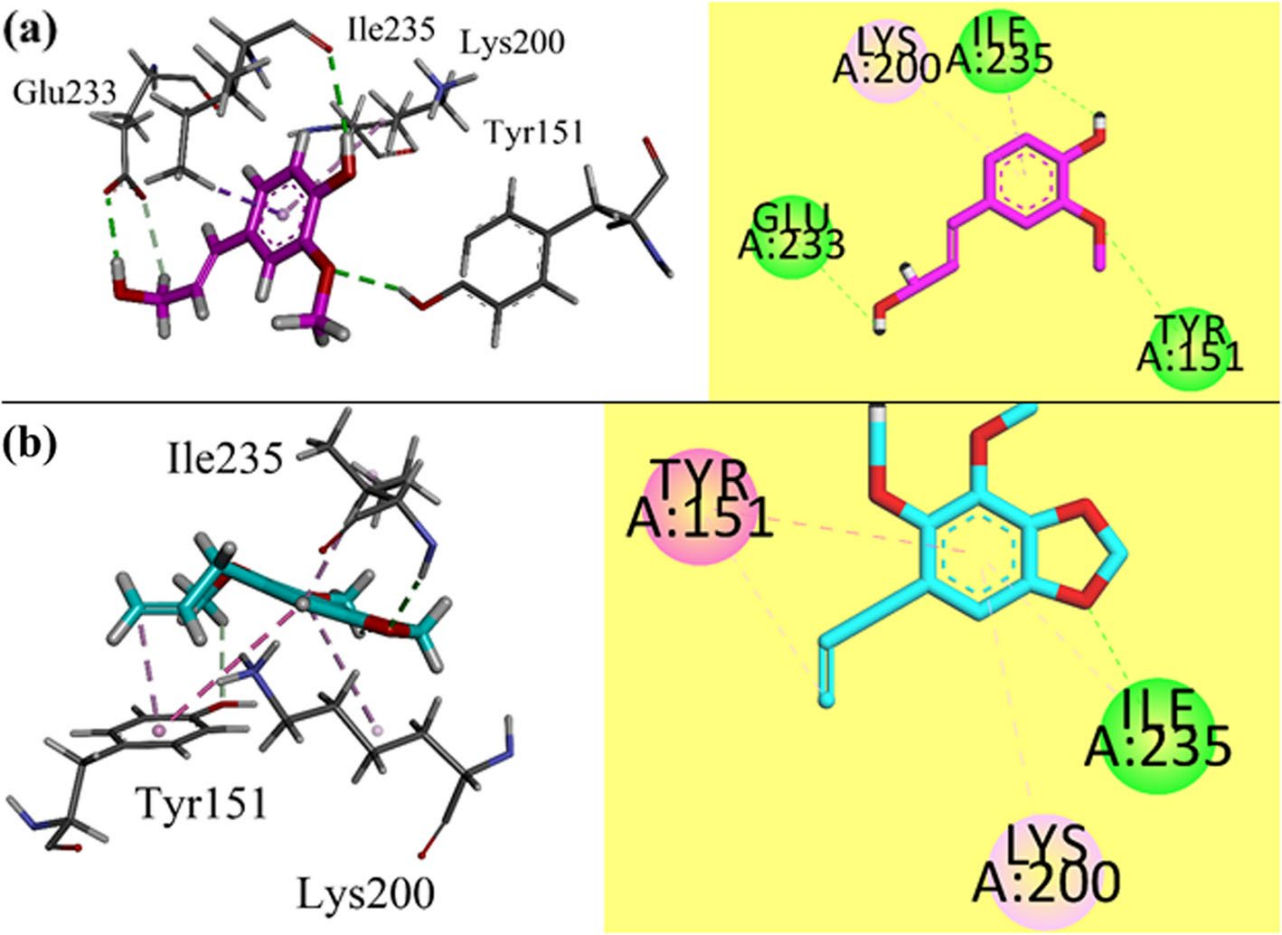
**a-b** 3-D / 2-D interaction plots of isolated compounds 1 and 2 respectively into the binding site of α-amylase (PDB ID = 4 W93)

Three-dimensional interaction plots of isolated compounds in the binding site of α-amylase are shown in Fig. 2a-b. Compound 1 showed three hydrogen bond interactions with Tyr151, Glu233 and Ile235. While Lys200 interacts via π-alkyl interaction (Fig. 2a). While compound 2 exhibited one hydrogen bond interaction with Ile235. Tyr151 interacts with the phenyl ring through π-π interaction (Fig. 2b). Computed binding energy values for compounds 1 and 2 in the binding site of α -amylase are − 5.8886 and − 6.3811 kcal/mol, respectively.

**Fig. 3:**
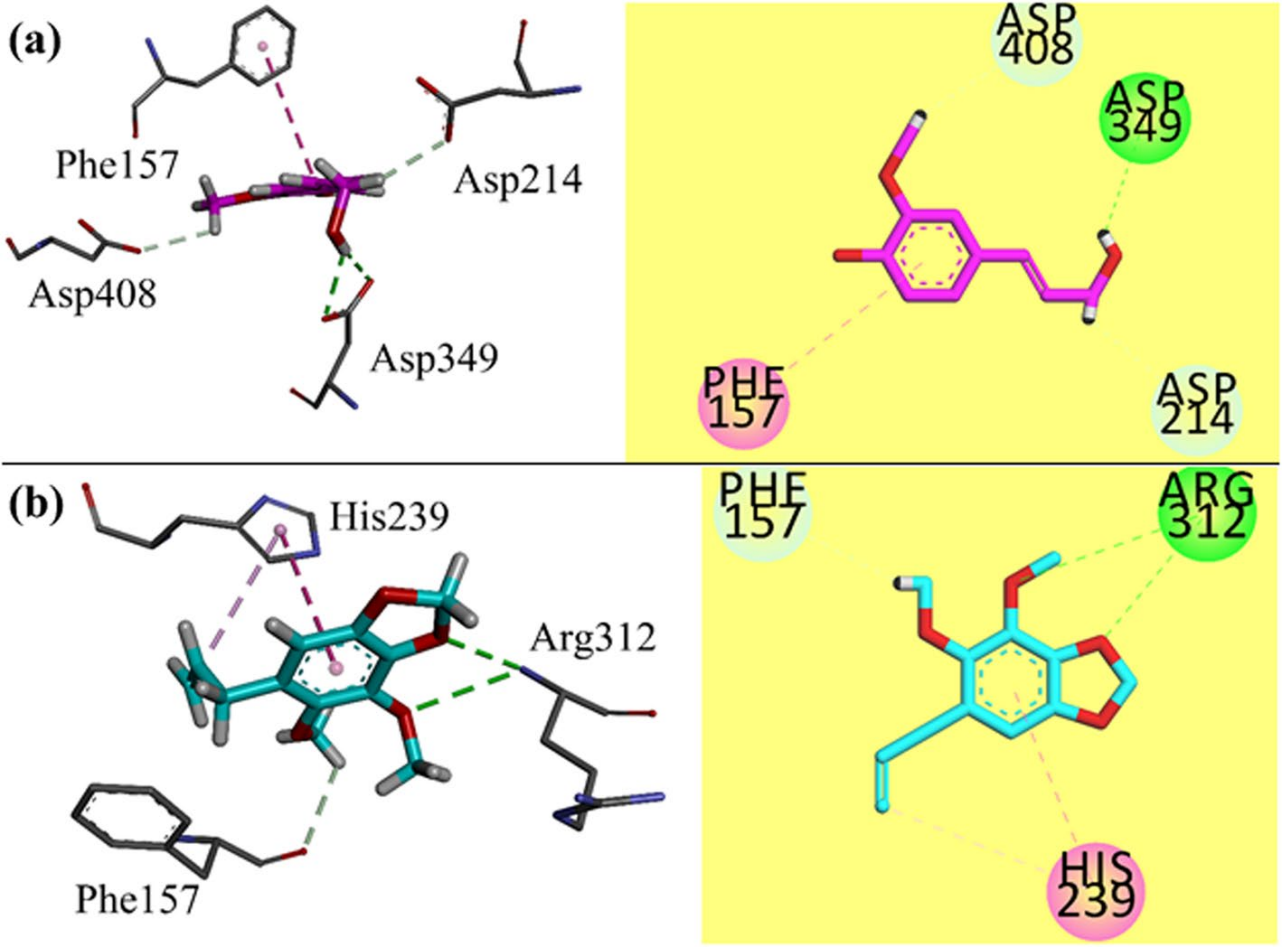
**a-b** 3-D / 2-D interaction plots of isolated compounds 1 and 2 respectively into the binding site of constructed α-glucosidase

Next, α-glucosidase, we performed docking studies of isolated compounds into the binding site of the constructed model from our previous studies. Three-dimensional interaction plots of isolated compounds in the binding site of α-amylase are shown in Fig. 3a-b. Hydroxyl group of compound 1 oriented itself toward Asp349 and established a conventional hydrogen bond interactions. While Phe157 forms π-π interaction with phenyl ring (Fig. 3a). While in compound 2, Arg312 formed a bifurcated hydrogen bond interaction with methoxy and dioxoleoxygen atoms. His239 forms π-π interaction with phenyl ring (Fig. 3b). Computed binding energy values for compounds 1 and 2 in the binding site of α -glucosidase are − 5.0339 and − 5.7104 kcal/mol respectively.

## Discussion

One of the most interesting fields of pharmacological research is the discovery and development of novel antioxidant drugs. Although oxygen is an essential component of aerobic life, it can have a negative impact on our health by causing the formation of reactive oxygen species (free radicals), which can lead to diseases such as diabetes, coronary heart disease, cancer, neurodegenerative disorders (Alzheimer’s and dementia), immune suppression, atherosclerosis, ulcers and aging [44]. Hydroxyl, nitric oxide, superoxide, and lipid peroxyl are the most frequent free radicals, while singlet oxygen and hydrogen peroxide are the most common non-free radicals [45, 46]. Nonetheless, defensive systems protect nearly all living beings from free radical damage, such as a protective antioxidant defense system that slows the production of free radicals as well as another system that creates chain-breaking antioxidants to scavenging and stabilizing free radicals [47]. When the rate of free radical production exceeds the capacity of the body’s defense mechanisms, however, severe tissue injury happens [48]. Consequently, drugs having potential free radical scavenging effects are beneficial in the prevention and treatment of different diseases [49]. Antioxidant chemicals are known to have biochemical effects through a variety of pathways, including chain initiation inhibition, metal ion chelation, peroxide breakdown, long-term hydrogen abstraction, reductive ability, and radical scavenging [16]. Hence, several techniques for determining antioxidant activity have been suggested. The DPPH method is most widely used protocol to assess the scavenging ability of free radicals of drugs [50]. Antioxidants scavenge DPPH radicals by donating hydrogen, resulting in decreased DPPH-H, which ultimately changes color from purple to yellow after reduction and which is quantified by analyzing absorbance of compounds at 517 nm wavelength [51]. The antioxidant capacity of the samples is used in the ABTS test to prevent the oxidation of ABTS to the ABTS^++^ radical cation [52].

The α-glucosidase and α-amylase assays are very common and well-known tests for determining the preliminary antidiabetic activity of an unknown compound [53]. The in-vitro antidiabetic potentials of the samples were determined using α-glucosidase and α-amylase assays. As this α-amylase enzyme was found in saliva and pancreatic juice, responsible for conversion of large polysaccharides into smaller molecule [21]. However, α-glucosidase was found in small intestine and responsible for breakdown of disaccharides into monosaccharides. The inhibitory action on α-amylase and α-glucosidase enzyme delays the metabolism of carbohydrate and reduces the postprandial blood glucose level [10, 21]. At this time, acarbose was the important drug of choice for the inhibition of enzyme to delay the metabolism of carbohydrate and it also reduces post prandial blood glucose level, but it has some adverse drug reaction like diarrhea and intestinal disturbances [4, 21]. If any compound from natural source has such kind of inhibitory effect on metabolic enzyme, it will receive high attention. Various natural products and synthetic compounds have been evaluated for the antidiabetic potentials. The beta cells have a vital role in diabetes. The excessive free radicals within the body damage the beta cells which ultimately complicate diabetes. Therefore, antioxidant is used as a supplementary target for the management of diabetes. The natural and synthetic sources have been explored for the discovery of new antioxidants. However, it is the need of the day to explore compounds which can treat diabetes targeting multiple sites. In this research, we have isolated compound 1 and 2 which are coniferol and dillapiole respectively. We compared the data of our isolated compounds with the published literature [54, 55]. To the best of our knowledge and literature survey, the antidiabetic of these compounds has not been previously reported. We explored both of these compounds for their potential antidiabetic activities.

The molecular docking is one of the reliable approaches to find out the interaction of a molecule with the binding protein for the management of various ailments [56–58]. We have performed docking studies to explore the possible binding orientation and the role of each isolated compound via their computed binding energy values. Binding orientations pattern suggested that compound interacted with the binding site residues of both the studied via hydrogen bond and hydrophobic interactions to stabilize ligand-enzyme complex. Computed binding energy values revealed that both compounds may have strong synergistic effects in lowering the blood glucose level.

## Conclusions

Based on our results, it can be assumed that the most of plant fractions that were examined herein have strong antioxidant capacity, which can be linked to the presence of high molecular weight phenolics. The plant also exhibits dose-dependent inhibitory action against alpha glucosidase and alpha amylase enzymes. Based on the relative potency of chloroform fractions, it was semi-purified to five different phyto-fractions CHF-1 to 5. The five phyto-fractions were relatively dominant in tested in-vitro targets. Among those, CHF-3 was dominant in activities due to the presence of bioactive compounds. The CHF-3 was purified to obtained compounds 1 and 2. The two compounds were excellent in in-vitro antidiabetic assays. Computed binding energy values of compounds 1 and 2 via docking studies revealed that both compounds may have strong synergistic effects in lowering the blood glucose level. The two compounds were also dominant in lowering the blood glucose levels in experimental animals. The study provides a baseline guideline for the use of crude extract and isolated compounds of *Allium consanguineum* for the management of diabetes.

## Data Availability

All data generated or analyzed during this study are included in this published article.

## Abbreviations

DPPH: 2,2-diphenyl-1-picrylhydrazyl
ABTS: 2,2′-azino-bis(3-ethylbenzothiazoline-6-sulfonic acid)
H_2_O_2_: Hydrogen peroxide
ADA: American Diabetes Association
T1DM: Type 1 diabetes mellitus
IDDM: Insulin-dependent diabetes mellitus
T2DM: Type 2 diabetes mellitus
NIDDM: Non-insulin-dependent diabetes mellitus
SEM: Standard error mean
